# Clinical experience with the artificial bone graft substitute Calcibon used following curettage of benign and low-grade malignant bone tumors

**DOI:** 10.1038/s41598-017-02048-w

**Published:** 2017-05-11

**Authors:** Joerg Friesenbichler, Werner Maurer-Ertl, Marko Bergovec, Lukas A. Holzer, Kathrin Ogris, Lukas Leitner, Andreas Leithner

**Affiliations:** 1 Department of Orthopedic Surgery, Medical University of Graz, Graz, Austria; 2 Institute for Forensic Medicine, Medical University of Graz, Graz, Austria

## Abstract

Artificial bone graft substitutes, such as Calcibon, are becoming increasingly interesting as they do not cause donor site morbidity which is an advantage compared to autologous bone grafts. The aim of this study was to evaluate the efficacy and potential complications associated with the use of Calcibon. Twenty-seven patients with benign and low-grade malignant bone tumors were treated with curettage and refilling of the bony cavity. Based on the radiological classification system of Neer, these lesions only comprised Grade I lesions, describing cysts that only require curettage and filling, but no additional treatment. At a mean follow up of six months we observed radiological consolidation without resorption of the bone graft substitute. These observations were also made at a mean follow-up of 13 and 32 months, respectively. According to the classification system of Goslings and Gouma we observed six surgical complications. Summing up, Calcibon seems to be a reliable bone graft substitute with low complication rates. However, delayed resorption should be expected. Calcibon seems to be an alternative to autologous bone grafts or allografts in adequate indications.

## Introduction

Benign bone tumors and low-grade malignancies are usually treated with intralesional resection or curettage. After curettage, the bone cavity can be left unfilled or it can be refilled with autologous or allogenic material^1, 2^. The final result should be a full bony ingrowth and a remodeling of the graft into mature bone.

A known side effect of autologous bone grafts originating from the iliac crest is donor side morbidity with risks like infection, delayed wound healing, neuro-vascular injuries, heterotopic ossification and local pain, which are the most commonly reported complication^2, 3^. As an alternative to autologous bone grafts, allogenic or artificial bone graft substitutes (ABGS), such as demineralized bone matrix, bone graft extenders, or bone morphogenic proteins, can be applied^2, 4–8^. However, allogenic materials have been reported to be immunogenicity and to transfer infectious diseases with complication rates for infection up to 12.2%^9, 10^. On the other hand, some bone void fillers in general have also been associated with higher complications rates up to 33%^1, 2, 5, 8, 11^.

Bone graft extenders like ceramics, salts, or synthetic products, such as polymethylmethacrylate (PMMA), are often used to refill bone cavities as they provide the advantage that they are moldable and can be used to fill irregularly shaped defects. What is more, ABGS are freely available without any restrictions of access, there is no reason for disease transmission.

Calcibon (Biomet, Warsaw, IN) is a synthetic bone graft substitute with osteo-conductive properties. It belongs to the family of α-tricalcium-phosphates (α-TCP). Calcibon is a chemical composition of 62.5% α-TCP, 26.8% dicalcium phoshate dihydrate, 8.9% calcium carbonate and 1.8% precipitated hydroxyapatite. In order to liquefy this mixture, 1% disodium hydrogen phosphate dodecahydrate has to be added with an ideal liquid-to-powder ratio of 0.35 mL/g^12^. However, a microporosity with a pore size of less than 1 μm is only present in 30 to 40% of the resulting graft^12^. Following its application to the cavity and drying, Calcibon’s biomechanical properties become similar to hydroxyapatite^13^. Due to its ability of bio-degradation and osteoconductive features, Calcibon is eventually getting reabsorbed by osteoclasts resulting in the remodeling of this grafting material into new and healthy bone. This has also been shown for other injectable calcium phosphate cements^12–14^. According to the manufacturer’s guidelines, the time until resorption of Calcibon depends on the area of application, as well as on the proximity to healthy and well-vascularized bone. Resorption of 22.9% of the cement volume used for kyphoplasty was observed by Maestretti *et al*.^15^ during a follow-up of more than 10 years.

The aim of this prospective, non-randomized study was to assess the efficacy of Calcibon regarding its resorption and remodeling into healthy bone following the curettage of bone tumors for a minimum follow-up of 12 months. Furthermore, complication rates were evaluated.

## Results

The study group consisted of 20 female and 7 male patients. The patients’ mean age at operation was 44 years (range, 17 to 72). Ten lesions were located in the proximal or distal tibia or fibula, two in the proximal and five in the distal femur, respectively. Three bone tumors were located in the proximal humerus, one lesion in the pubic bone and six lesions in the fingers. The lesions comprised fifteen enchondromas, five low-grade chondrosarcomas (G1), three aneurysmatic bone cysts and four simple or juvenile bone cysts. According to the bone healing classification system of Neer, all lesions were graded as grade I, which indicates that curetted cysts require no further treatment. There were no grade II, III or IV lesions. However, in six cases an additional plate osteosynthesis was required for stabilization based on an intraoperative decision of the surgeon.

**Figure 1:**
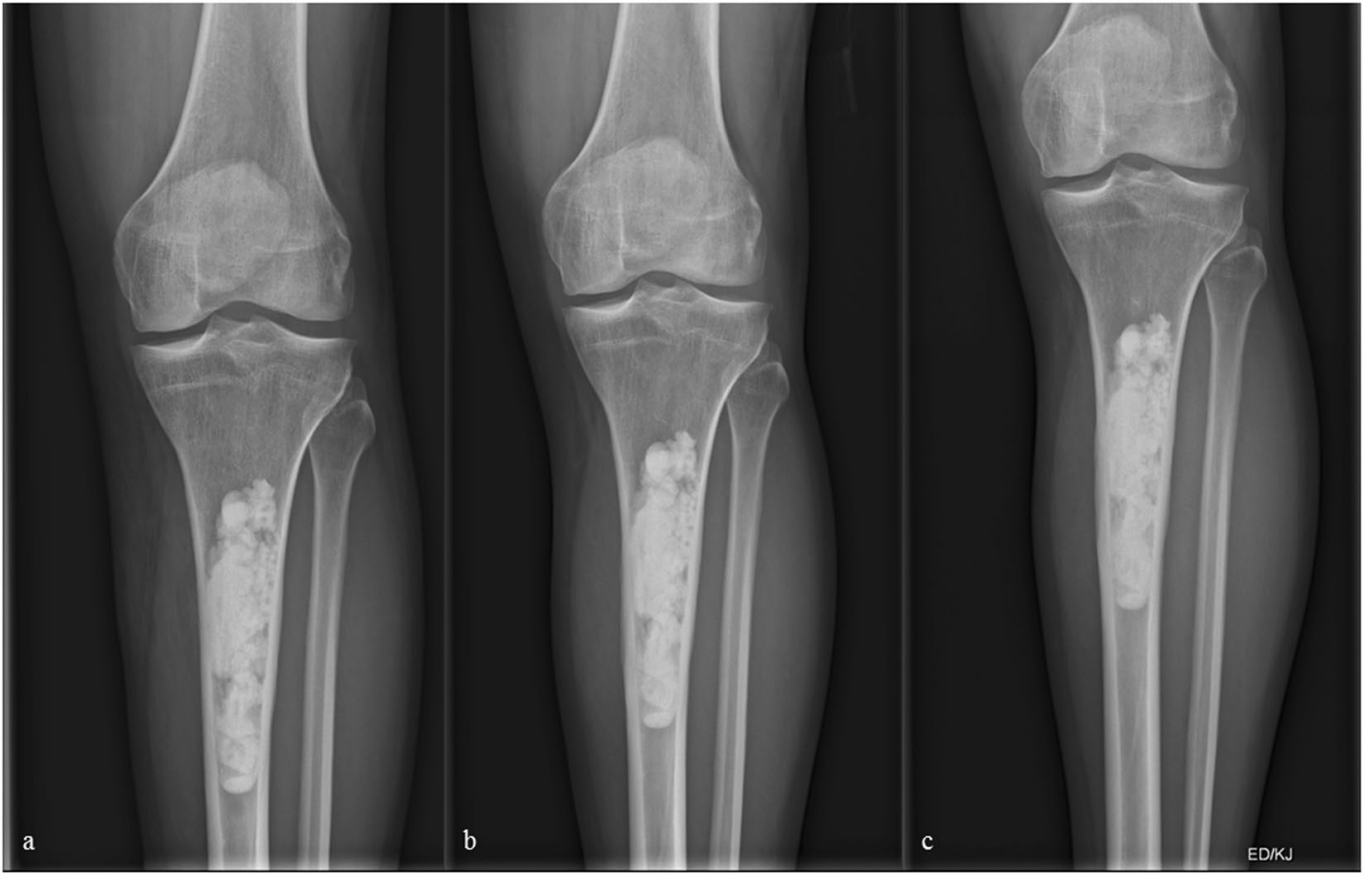
(**a**–**c**) X-ray of a 36 year-old male patient few days following curettage of a low-grade chondrosarcoma of the left proximal tibia. (**b** and **c**) Follow-up radiographs 7 and 13 months following index surgery showing integration but no resorption of the artificial bone graft substitute.

**Figure 2:**
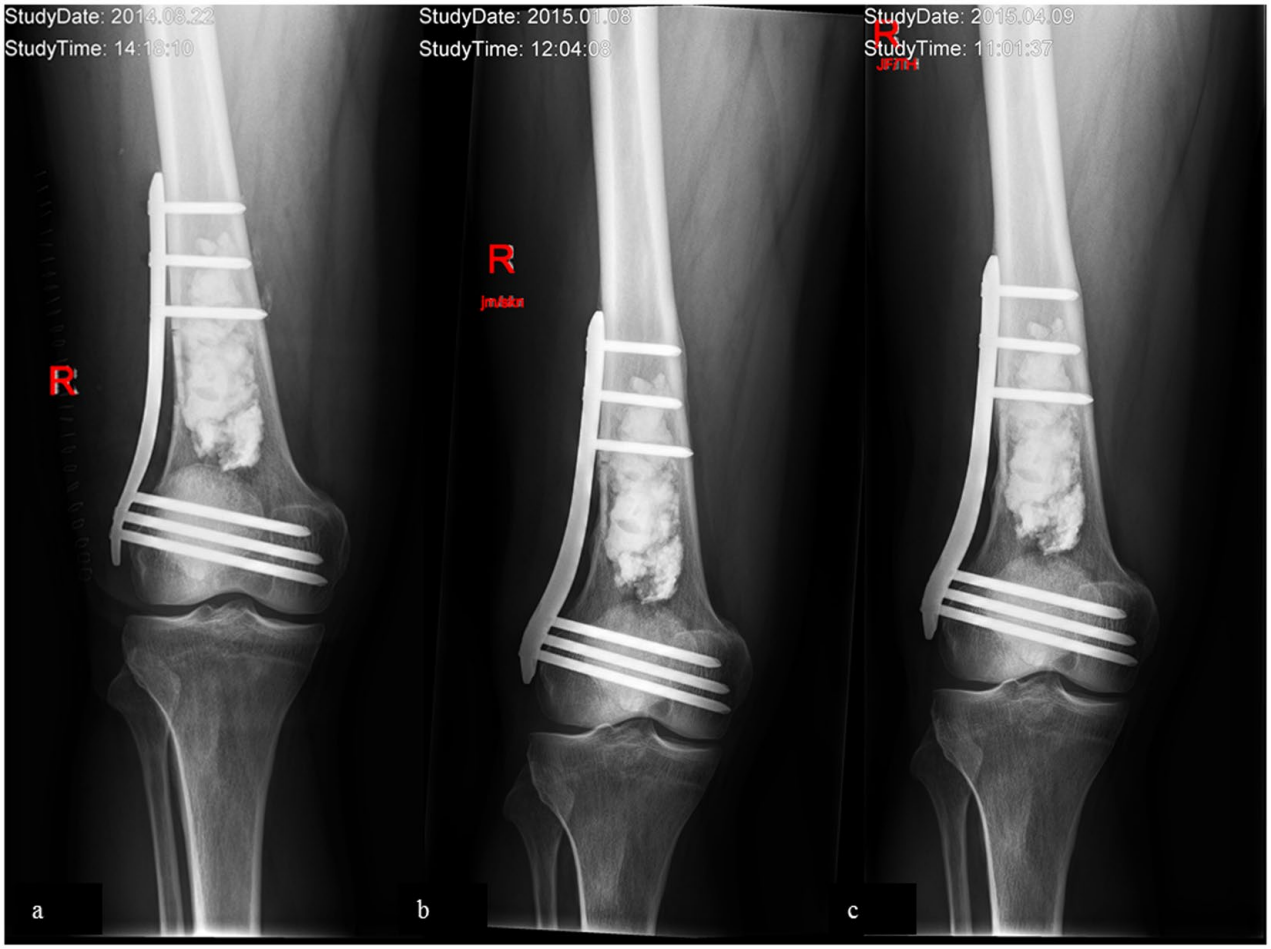
(**a**–**c**) X-ray of a 64 year-old male patient few days following curettage of an enchondroma of the right distal femur. To enhance primary stability, a plate osteosynthesis was done. (**b** and **c**) Follow-up x-rays 5 and 11 months following index surgery showing integration but no resorption of the bone graft substitute.

The mean amount of Calcibon used for refilling of the cavities was 23 ml (range, 10 to 60, Figs 1a and 2a). In the case of a low-grade chondrosarcoma located in the proximal tibia and the distal femur, and in another case of a juvenile bone cyst situated in the proximal humerus, we had to mix Calcibon with β-TCP Vitoss (Stryker, Kalamazoo, MI) in order to refill the big resulting bony cavities.

The average postoperative follow-up for all patients was 26 months (range, 1 to 50). One patient was lost of follow-up one month after surgery.

At a mean follow up of six months (range, 1 to 9) we observed radiological consolidation following curettage in 23 patients. However, the ABGS Calcibon had not been resorbed by that time (Figs 1b and 2b). These observations were in line with our results obtained at 13 (range, 10 to 16, n = 19) and 32 (range, 18 to 50, n = 19) months of clinical and radiological follow-up (Figs 1c and 2c).

We observed no local recurrences during the follow-up periods indicated.

### Complications

One patient developed idiopathic postoperative femoral nerve palsy following curettage of an enchondroma located in the distal femur. Thorough clinical examinations, which included MRIs of the brain and the lumbar spine, as well as examinations of peripheral nerve conduction velocities, did not yield pathological results and thus were not able to sufficiently explain the reason for this palsy.

Another patient suffered a traumatic fracture of the distal femur after falling over one month after primary surgery. This fracture required revision of the plate osteosynthesis which had been used for stabilization after curettage. Furthermore, one patient presented with delayed wound healing of the proximal fibula. Though aseptic, this complication required revision and a removal of the bone graft substitute two months after initial surgery.

In two other cases the removal of osteosynthesis plates of the proximal and the distal femur was indicated due to persistent pain nine and thirteen months following stabilization. In another patient we observed an extravasation of Calcibon in postoperative x-rays following surgery of the proximal fibula. This was of no further consequence as the patient was free of symptoms.

When being classified according to the classification system of Goslings and Gouma^16^ the complications described above can be summarized as follows: one patient suffered a grade 0 (no harm), another one a grade I complication, the latter referring to meaning a temporary disadvantage which does not require surgery. Four patients presented with grade II complications showing total recovery after revision. No patients suffered grade III, IV, or V complications.

## Discussion

The current study demonstrates that the ABGS Calcibon shows rapid bone consolidation, incorporation and durability, combined with a low complication rate. Further advantages of Calcibon lie in the possibility of a minimal invasive, percutaneous, transcortical administration like in case of vertebroplasty or kyphoplasty. This technique might also be used for be benign cystic bone lesions. The resilience of Calcibon, which is similar to hydroxyapatite and develops shortly after application and drying, theoretically allows an unrestricted postoperative mobilization, though restrictions may apply in individual cases. Overall, it can be concluded that ABGS Calcibon seems to provide a safe and effective long-term treatment option/reconstruction tool following the curettage of benign and low-grade malignant bone tumors. Its capability of resorption forms another advantage which suggests that Calcibon should be preferred as compared to PMMA, although resorption might take more than a decade which has been shown in the study of Maestretti *et al*.^15^.

One reason for delayed resorption might be the development of a membrane between bone and the ABGS but in order to prove this theory, tissue samples have to be taken what could be in the focus of further studies. Nevertheless, increasing knowledge about time till resorption could be beneficial in counseling patients and in advancing or restricting activities.

Complications directly associated with the ABGS Calcibon could not be observed in the current series. All revisions conducted were due to delayed wound healing or mechanical complications.

Depending on a surgeon’s preference, there are different approaches regarding the reconstruction of bony cavities following curettage. Refilling the curetted bone is known to enhance primary stability and acceleration of bone healing, whereas similar clinical results have been reported for unfilled defects^2, 5, 8, 11, 17^.

Complications and shortcomings associated with the usage of autologous and allogenic bone grafts have stimulated interests in potential alternatives, such as ABGS^2, 18, 19^. The use of TCP and fluid cements has yielded good and excellent results in spinal surgery which was performed for fracture stabilization, such as vertebro- or kyphoplasty^15, 20–22^.

On the other hand, high complications rates of up to 33% have been reported for various bone graft extenders^1, 2, 5, 8, 11^. Pain, aseptic inflammation and formation of reactive soft tissue-cysts are amongst the most commonly reported complications^1, 7, 23^. Extravasated liquid or semi-liquid bone graft extenders have been discussed as most likely causes of these.

Recently, Kaczmarczyk *et al*.^24^ reported a minimally invasive treatment of cystic bone tumors with a bi-phasic, injectable ceramic bone substitute (CERAMENT, BoneSupport, Sweden) which yielded good results after one year of follow-up showing a nearly total osseous consolidation. Similar observations were made in the current series. Even though osseous consolidation was observed soon after surgery, the bone graft substitute Calcibon was not fully resorbed until the end of our follow-up period, which indicates a delayed resorption profile of this substance. Nevertheless, we did not observe any serious complications associated with this material.

Van Hoff *et al*.^2^ related a retrospective study assessing the clinical outcome of surgical management of benign bone tumours treated with ultraporous β-TCP morsels. At 6 months of follow-up 50% of the ABGS had progressed to trabeculation. One advantage of this study was the rapid resorption of the β-TCP morsels with low complication rates. On the other hand, filling irregularly shaped defects by using morsels is aggravated and compared to pastes the bone window has to be bigger with reduction of the stability of the effected bone.

One limitation of the study is the short mean follow-up period of 26 months, which was not sufficient to document a full resorption of the ABGS Calcibon. In order to illustrate this material’s resorption profile a longer follow-up period is required. Nevertheless, it could be shown, that there was a low complication rate by using this bone void filler. In line with our findings, Van Lieshout *et al*.^13^ and Maestretti *et al*.^15^ related resorption of Calcibon to require more than 10 years after spinal surgery.

Like in other series reporting the experiences and the clinical results of bone graft extenders, statistical analysis is lacking, which is another limitation. Furthermore, the study population was too small and too heterogeneous, therefore, the data could not be analyzed sufficiently.

In conclusion, this is the first series reporting Calcibon as bone void filler following curettage of benign and low grade malignant bone tumors. This ABGS seems to be a reliable solution for defect management with low complication rates. However, it seems to require a longer period of time until resorption which has also been shown in case of spinal surgery previously. In summary, we think that Calcibon can be recommended as an alternative to autologous bone grafts or allografts in suitable indications.

## Methods

Between June 2013 and February 2015, we treated 27 patients with benign and low-grade malignant bone tumours with curettage and refilling of the bony cavities using the artificial bone graft substitute Calcibon. Patients with high-grade malignancies were excluded. Clinical follow-ups and two-planar x-rays were performed 6 weeks, 3 months, 6 months and one year after surgery. All operations were performed by experienced orthopaedic tumor surgeons (AL, WME, MB).

After surgical exposure of the affected bone, an osseous window was created through which the lesion could be accessed. Curettage was performed under radiographic control using a c-arm. The curetted tissue was sent for histopathologic examination. Calcibon was prepared according to the manufacturer’s instructions, injected into the void and allowed to dry for several minutes. If possible the osseous lid was re-apposed and the layers of the wound were closed.

Time required for osseous consolidation, local recurrence rates, and complications were evaluated. Complications were evaluated according to the classification system of Goslings and Gouma^16^ as follows: 0 (no harm), 1 (temporary disadvantage, no reoperation), 2 (recovery after reoperation), 3 (permanent damage/disability), 4 (death), and 5 (unclear as a result of untimely death).

Osseous consolidation was classified according to the modified Neer score^24, 25^.

### Ethical approval and informed consent

The study was a nonregistered single-center investigation and was approved by the Ethics Committee of the Medical University of Graz (EK 29-015 ex 16/17). All methods were performed in accordance with the relevant guidelines and regulations. Written informed consent was obtained from all patients.
